# FOIM: Thermal Foaming of Shape Memory Polyurethane Foil

**DOI:** 10.1002/marc.202401103

**Published:** 2025-02-06

**Authors:** Anna‐Lisa Poser, Thorsten Pretsch

**Affiliations:** ^1^ Department of Shape Memory Polymers Fraunhofer Institute for Applied Polymer Research IAP Geiselbergstraße 69 14476 Potsdam Germany; ^2^ Department of Polymer Chemistry Institute of Chemistry University of Potsdam Am Neuen Palais 10 14469 Potsdam Germany

**Keywords:** highly compressible, one‐way shape memory effect, polyurethane foam, programmable material, shape memory polymer, semi‐finished product, thermo‐responsive

## Abstract

This work introduces the semi‐finished product FOIM, a neologism from *FOI*l and foa*M*, a phase segregated polyester urethane urea (PEUU) foam, which is synthesized from poly(1,6‐hexylene adipate) diol, 4,4’‐methylene diphenyl diisocyanate, polyethylene glycol and water as blowing agent. The PEUU is obtained by following a one‐shot synthesis and is characterized by a hard/soft segment ratio of 1.06 and an open pore content of 78%. Differential scanning calorimetry reveals a melting peak at 45 °C and a crystallization signal at 14 °C, both of which are associated with the phase transitions of the soft segment. The glass transition temperature, which is determined as a local maximum within the tan δ using dynamic mechanical analysis, is 1 °C. During programming, the foam is heavily compressed at 60 °C. Once unloaded at 23 °C, a translucent foil, the FOIM, is obtained. When reheated to 60 °C, the foil switches back to the foam due to its pronounced shape memory properties. Intriguingly, the structure remains largely unaffected by the drastic deformation as indicated by buoyancy and heat transmission measurements. The ease of manufacture and functionality makes the technology attractive for applications, in which both low transport volumes and drastic shape changes are desired.

## Graphical abstract

This study introduces FOIM, a polyester urea urethane foil that can be foamed once if it is heated to 60 °C because of its pronounced shape memory properties. The ease of manufacture and functionality makes the technology attractive for applications, in which both low transport volumes and drastic shape changes are desired.

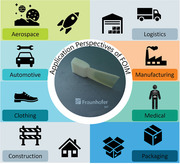

## 1 Introduction

Shape memory polymers (SMPs), which are categorized as smart materials,^[^ ^1^ ^]^ undergo a shape change in response to an external stimulus, most commonly an increase in temperature. Once thermomechanically treated, so‐called programmed, SMPs can be stabilized in a temporary shape and respond autonomously to a change in temperature by largely returning to their original shape.^[^ ^2^ ^]^ Phenomenologically speaking, the one‐way shape memory effect (1W SME) after programming is driven by entropy elasticity.^[^ ^3^ ^]^

Polyester urethane ureas (PEUUs) are block copolymers consisting of a soft segment phase made up of polyols, e.g. polyester diols, and a hard segment phase composed of diisocyanates and diols as chain extenders. Today, polyurethane foams are widely used in insulation and upholstery applications, and they also have the advantage of ensuring good pressure distribution as a material for insoles.^[^ ^4^ ^]^ A pronounced physical or chemical cross‐linking and a high degree of phase segregation are commonly regarded as prerequisites for distinct shape memory properties, including good shape fixity and shape recoverability.^[^ ^5^ ^]^ From a mechanistic point of view, the stabilization of a deformed PEUU in a temporary shape is enabled by both soft segmental crystallization and/or vitrification. In turn, soft segmental devitrification and melting can be used to unblock the elastic shape recovery force, whereupon the material mostly returns to its original shape.^[^ ^6^ ^]^

The 1W SME has been reported for solid polymeric materials as well as for porous polymeric materials like foams.^[^ ^7^, ^8^, ^9^ ^]^ SMP foams offer the advantage of a large volumetric change, which is attractive in industries, where the transportation volume should be kept as small as possible. For this reason, initial research activities were launched in space technology at the turn of the millennium.^[^ ^8^, ^10^, ^11^ ^]^ Indeed, in 1999 the concept of “cold hibernated elastic memory” (CHEM) for open cellular polyurethane (PU) structures was introduced by Sokolowski et al. against the background of applications in aerospace.^[^ ^10^ ^]^ At a later point in time, almost complete shape recoverability was witnessed after storing a compressed PU foam for two months at temperatures below the glass transition temperature of the material, thus proving the possibility of controlled awakening of the polymer from hibernation at a predefined point in time.^[^ ^8^ ^]^ Later research also demonstrated outstanding benefits of thermally deployable polymeric foams in medical applications, such as the filling and occluding of aneurysms.^[^ ^9^, ^12^, ^13^ ^]^ Apart from that, highly compressible PU foams have been thermomechanically investigated, at which deformations of up to 93.4% have been applied in course of programming.^[^ ^14^ ^]^

However, a frequently discussed aspect of standard PU foam production is the significant health risk in the workplace. Especially, directly after preparation, PU can contain free isocyanate groups, which are known to pose a health risk to people who handle them. Isocyanates have a sensitizing effect when inhaled or touched, irritate the eyes, skin, and respiratory tract, damage the liver and kidneys under repeated exposure, and are even suspected of causing cancer.^[^ ^15^ ^]^ To reduce those risks there is a requirement for a sufficient time frame, which needs to pass before the freshly synthesized materials can be handled securely, as the isocyanate groups need to react with moisture in the environment first, forming less harmful amines. This applies to both the technical manufacturing on production plants as well as the on‐site production of PU foams using aerosol cans or pressurized containers in terms of one‐component (1C) and two‐component (2C) manufacturing.^[^ ^16^ ^]^ On‐site production is particularly widespread in the construction industry, where PU foams are used for installing windows and doors, sealing joints, bonding, and insulation.

**Figure 1:**
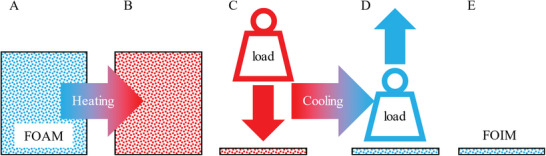
Scheme of programming PEUU foam to foil (FOIM), A) starting with the foam at room temperature followed by B) heating above the melting transition temperature of the switching segment, C) strong compression, D) cooling below the recrystallization temperature range of the switching segment and E) unloading.

To follow a simple strategy regarding the handling of PU foam, we herein introduce FOIM (*FOI*l + foa*M*), a foil‐like semi‐finished product prepared from self‐synthesized shape memory PEUU foam characterized by high compressibility. Severe deformation as central part of thermomechanical treatment (programming) including cooling to solidify the switching segment of the polymer, as depicted schematically in **Figure** **1**, ensured that the obtained foil (FOIM) is in a stable state at room temperature.

In this work, it will be demonstrated that upon triggering the 1W SME, “thermal foaming” occurred, resulting in the recovery of large parts of the original foam. The synthesis of the foam, the technical boundary conditions for its programming, and the shape recovery behavior will be investigated. As a useful side effect, it will be demonstrated that no free isocyanate groups are present in the foil (FOIM), and that gradual shape recovery allows a fine‐tuning of the material's mechanical properties, e.g. on‐site, by recovering it into a predefined shape, where e.g. ball rebound properties depend on the degree of shape recovery.

## 2 Results and Discussion

The foamed PEUU was synthesized by means of the one‐shot method.^[^ ^17^ ^]^ It was obtained within 3 to 5 s after addition of the 4,4’‐methylene diphenyl diisocyanate (MDI) to the reaction mixture, which contained poly(1,6‐hexylene adipate) diol (PHA) (M_n_ = 2970 g mol^−1^) as polyester polyol and the cross‐linker polyethylene glycol (PEG) (M_n_ = 200 g mol^−1^). The foam was characterized by a density of 80 kg m^−3^, which according to DIN EN ISO 33861 qualifies it as a low‐density foam with the lower limit for a high‐density foam being 250 kg m^−3^.^[^ ^18^, ^19^ ^]^ The foam displayed poor solubility in N,N‐dimethylformamide (DMF), with only 12.1% by weight dissolving, indicating pronounced chemical cross‐linking.

**Figure 2:**
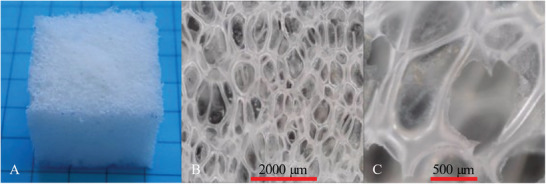
PEUU foam A) as a cubed‐shaped sample and B) & C) microscopic images at different resolutions.

Visual inspection of the foam (cf. **Figure** **2**A) showed an even distribution of cells. The cells were oriented in the direction of foaming, i.e. they were not completely circular but slightly oval, which was confirmed by microscopic imaging (cf. Figure 2B,C). The diameter of the cells ranged from 500 to 2000 µm. As only a few larger cells were detected, it was assumed that a rapid and almost complete coupling reaction had taken place. Commonly, a slow reaction of diisocyanate, polyester diol, and polyether diol favor the formation of larger cells due to the merging of multiple small and unstable cells.^[^ ^20^, ^21^ ^]^ As demonstrated by buoyancy measurements, 78% of the cells were open, which is desirable to ensure strong deformability during later programming without significant structural damage.

**Figure 3:**
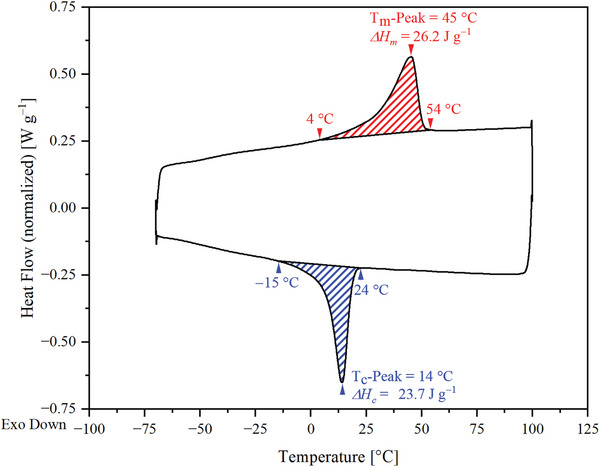
DSC thermogram of PEUU foam during second heating (in red color) and cooling (in blue color) at a rate of 10 °C min^−1^.

The thermal properties of the foam were characterized by differential scanning calorimetry (DSC) (**Figure** **3**). Two characteristic phase transitions became apparent in the second heating and cooling cycle. First, a broad phase transition ranging from 4 to 54 °C with a peak located at 45 °C occurred, which was assigned to the melting of the PHA soft segment, followed by a phase transition spreading from 24 to −15 °C with a peak located at 14 °C, which was assigned to the recrystallization of PHA.

Compared with the pure polyester polyol (Figure S1, Supporting Information), a decrease in the melting peak temperature by 10 °C and a reduction in the melting enthalpy from 92.1 to 26.2 J g^−1^ was observed while the crystallization temperature decreased by 25 °C and the enthalpy of crystallization was reduced from 89.0 to 23.7 J g^−1^. The less pronounced affinity of PEUU for crystallization is consistent with that of a similar PEUU, which was recently synthesized by our group and had a comparable content of hard segments.^[^ ^21^ ^]^ It was likely caused by chemical cross‐linking, resulting in the formation of a network structure.

**Figure 4:**
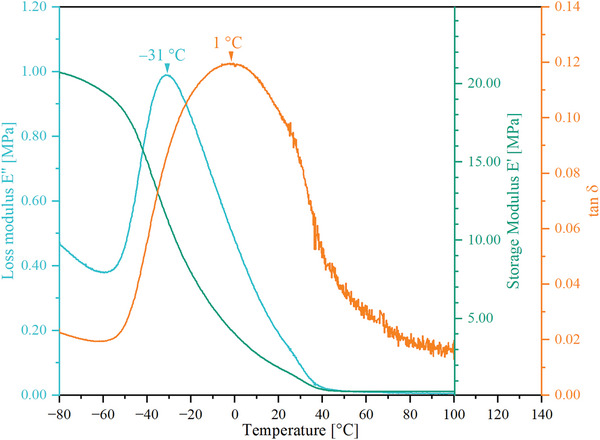
Determination of the thermomechanical properties of the pristine PEUU foam using DMA.

To further investigate the thermal properties of the PEUU, a dynamic mechanical analysis (DMA) measurement was performed. Herein, maxima of the loss modulus E’’ at ≈−31 °C and the loss factor tan δ at 1 °C were determined, indicating a glass transition (cf. **Figure** **4**).

**Figure 5:**
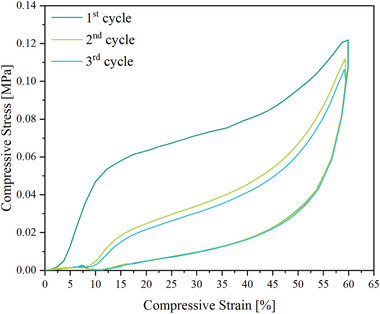
Cyclic stress–strain behavior of PEUU foam at 23 °C using loading and unloading rates of 50% min^−1^.

In the next step, the mechanical properties of the foam were examined. The CC_40_‐value was determined at 23 °C to evaluate the stress‐deformation characteristics in compression. With a CC_40_‐value of 70.6 ± 3.6 kPa, the foam exhibited a strong resistance to deformation (cf. Figure S3, Supporting Information).^[^ ^22^ ^]^ A similar measurement, but with a compression of 60% and a loading rate half as fast, was conducted to characterize the compressive stress–strain behavior in three consecutive cycles (**Figure** **5**). Elastic material behavior was demonstrated under cyclic compression.

**Figure 6:**
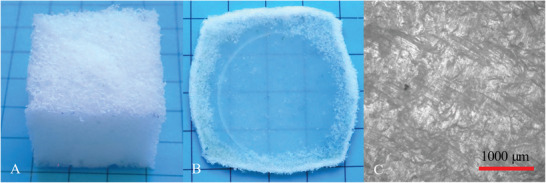
Shape memory PEUU foam: A) Cubed‐shaped sample with a size of 30 mm × 30 mm × 30 mm, B) the same sample after programming to a translucent foil, and C) microscopic image of the foil.

To further characterize the foam, the shape memory properties of a cuboid sample having a lateral length of 30 mm (cf. **Figure** **6**A) were investigated. The sample was heated to 60 °C, a compressive stress of 27.3 MPa was applied and the sample cooled to 0 °C to fix it in its temporary shape. The result was an ≈2.5 mm thin foil, which was characterized by stability at room temperature and a translucent appearance in its center part (cf. Figure 6B). The material behavior is reminiscent of that of a thermoplastic polyurethane vitrimer, which changes from opaque to transparent when strongly stretched,^[^ ^23^ ^]^ whereas such material behavior was also observed for poly‐ε‐caprolactone (PCL) with a molecular weight of 80 000 g mol^−1^ after deformation below its melting transition temperature.^[^ ^24^ ^]^ In course of deformation of PEUU foam, the material was pressed outward at the edges of the cuboid. Microscopic investigation of the programmed sample revealed that even at the edges no cells were observable any longer (cf. Figure 6C).

**Figure 7:**
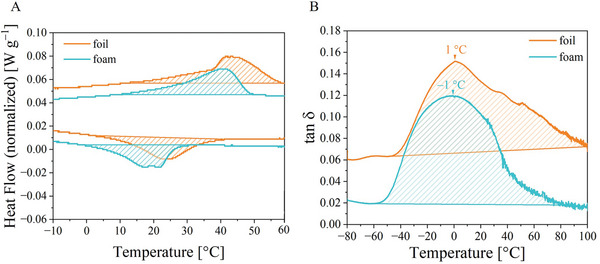
Characterization of pristine PEUU foam (in turquoise color) and programmed PEUU foil (in orange color): A) DSC thermograms showing the first heating and cooling at a rate of 1 °C min^−1^ and B) evolution of tan δ signal in DMA.

Due to the transparency of the material, it was first assumed that the switching segments tended to be vitrified, so that the crystallization of switching segments only took place to a minor extent. To elucidate the shape fixation mechanism, the programmed foil was quickly cooled to −80 °C in a DSC measurement. During subsequent heating, a broad melt transition was detected that was assigned to the switching segments (**Figure** **7**A). This indicates that crystallization contributed to the mechanism of shape fixation of the highly compressed foam. As shown in a DMA measurement on the programmed foil, the tan δ peak was almost unaffected, but the signal was extended to higher temperatures compared to the pristine foam (Figure 7B). This means that the glass transition might have had an influence on the fixation of the temporary shape. This assumption is supported by the fact that phase transition temperatures can increase with pressure as known e.g. from polycarbonate, polyethersulfone, and PCL.^[^ ^25^ ^]^ For these reasons, both the crystallization and the vitrification of the switching segments may have supported the good shape fixation.

**Figure 8:**
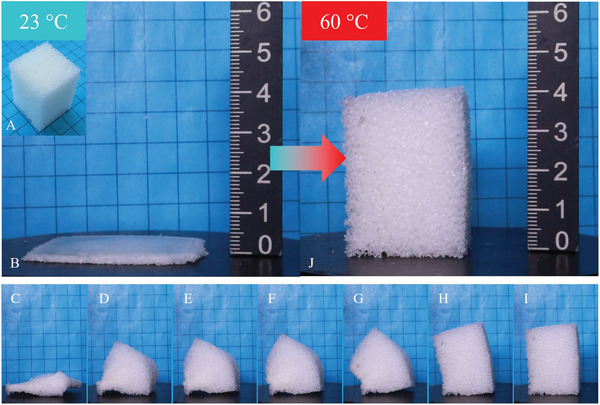
Thermoresponsiveness of PEUU foil: A) Permanent shape (foam), B) programmed shape (foil) and shape recovery behavior upon exposure to 60 °C in a heating chamber after C) 4 min, D) 8 min, E) 12 min, F) 16 min, G) 20 min, H) 24 min, I) 28 min, and J) 34 min.

Most importantly, the 1W SME was triggered upon reheating the foil to 60 °C. In the case of a sample, which measured 27.9 mm × 28.3 mm × 40.7 mm in its pristine state, this resulted in a significant expansion from 2.5 mm, characterizing its compressed, programmed shape, to 40.5 mm. Therefore, an increase in height by ≈1600% (**Figure** **8**) and a shape recovery ratio of 99.5% were verified.

Additionally, buoyancy measurements were carried out to estimate the open cell content of the material before and after programming. In its pristine state, the content of closed cells was determined to be 22% ± 0.8%. After programming and shape recovery it was slightly reduced to 17% ± 7.0%. The fact that closed cells were still present suggests that the foam structure was largely restored. A plausible explanation for the detected material behavior is that a considerable amount of gas escaped from open pores during compression, while the gas stored inside the closed pores was released through the walls of the pores by diffusion processes without building up pressure inside the cells. Since compression was carried out at 60 °C, at which the soft segments of the PEUU were amorphous, an enhanced air permeability to gas is assumed, so that only a small proportion of closed pores burst. Therefore, the foam with its high content of open pores exhibited a different material behavior than closed‐cell thermoset polyvinyl chloride shape memory foam, in which closed cells were converted to open cells due to compression during programming.^[^ ^25^ ^]^

Further evidence for the restoration of the original foam structure was provided by thermal conductivity measurements and by ball rebound tests. Indeed, when placed on a heating plate at a temperature of 100 °C, after 30 min the surface temperature of the pristine PEUU foam was 48.3 ± 2.7 °C and that of the recovered sample 48.9 ± 4.1 °C, indicating comparable thermal conductivities. In addition, the pristine foam exhibited a ball rebound of 19.1% in relation to the drop height. After programming to a foil, this value increased to 34.7%. When triggering the 1W SME by heating to 60 °C a rebound value of 19.7% was measured at room temperature. This result again indicates that the original foam structure was largely restored. In addition, step‐by‐step triggering of shape recovery enabled an adjustment of ball rebound height. In fact, when increasing the temperature in stages, progressive foaming was accompanied by a steady decrease in ball rebound height from 33.4% ± 5.0% (50 °C) to 28.2% ± 4.2% (100 °C) (cf. Figure S4, Supporting Information). This material behavior thus provides an indication of gradually adjustable property profiles. Overall, the combination of the conducted experiments indicates that the structural integrity of the PEUU foam remained intact throughout programming and shape recovery.

**Figure 9:**
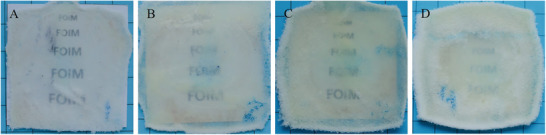
Programmed samples of PEUU foam using a compressive stress of 15.6 MPa. The height of the pristine samples was A) 10 mm, B) 20 mm, C) 30 mm, and D) 50 mm.

Next, the influence of sample height upon the transparency of programmed samples was examined (cf. **Figure** **9**). As expected, the transparency of the foil decreased steadily with increasing height of the foam samples. While the sample, which was originally 10 mm high (Figure 9A), was programmed into a clear, almost fully transparent piece of foil, which allowed a free view of the underlying writing, the samples, which were originally 20 and 30 mm high (Figure 9B,C) were less transparent after programming, but still translucent, and the underlying writing appeared blurred. A foil obtained by programming a 50 mm high sample (Figure 9D) was translucent, but the transparency disappeared. When handling the materials, it also became clear that the flexibility and stiffness can be altered by changing the thickness of the foam. This can be particularly useful when considering applications, such as transparency in packaging and flexibility when the foil needs to be bent.

**Figure 10:**
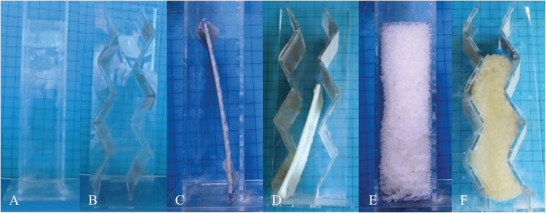
Cavity structures consisting of A) a cuboid shape (unfilled), B) a serrated shape (unfilled), C) the cuboid with a sample of FOIM at 23 °C, D) the serrated shape with another sample of FOIM at 23 °C, E) the cuboid after heating FOIM to 60 °C and F) the serrated shape after heating FOIM to 60 °C. There is centimeter paper in the background to allow an estimation of the sizes.

Larger samples measuring 120 mm × 50 mm × 40 mm were produced to investigate the scalability of the programming approach. This time, a compressive stress of 5 MPa was applied at 60 °C to the samples. The resulting thermally switchable FOIM, was then placed in cavities with different geometries (**Figure** **10**).

Indeed, both a cuboid shape and a cavity structure with two serrated walls facing each other were filled, when placing FOIM inside of them (Figure 10B,C) and heating the samples to 60 °C (Figure 10E,F). In the second case, it becomes obvious, that the material almost completely foams out the complex shape, which makes it an attractive insulation technology for use in geometrically demanding environments.

Before and after foaming, FT‐IR spectra were recorded to determine whether free isocyanate groups were present in the foam (Figure S5, Supporting Information). In fact, no signal occurred in the carbonyl stretching region between 2280 and 2240 cm^−1^ as associated with asymmetric stretching vibrations of unreacted isocyanate groups.^[^ ^26^ ^]^ As expected, stretching vibrations of free and hydrogen‐bound carbonyl groups appeared instead as a dominating signal and a broad shoulder in the FT‐IR spectra at 1750 and 1700 cm^−1^, respectively.^[^ ^27^ ^]^ This implies that the approach does not pose a health risk to people carrying out thermal foaming when working with FOIM.

However, chemical mixtures such as those released from pressure vessels for the manufacture of construction foams also have an advantage over FOIM. They react chemically with their environment and thus ensure a certain degree of adhesion to material surfaces in their immediate vicinity. Against this background, the user must decide whether a high level of adhesion is required. FOIM, on the other hand, offers the prospect that the material can be removed again without leaving any residue, as essentially only physical interactions with the environment can be assumed.

**Figure 11:**
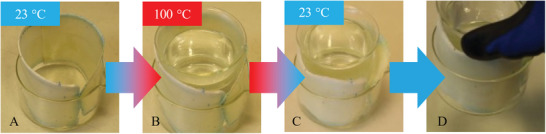
FOIM is used as joining technology: A) Placing of FOIM in a 150 mL crystallizer bowl; B) insertion of a 120 mL beaker filled with 100 °C hot water to trigger the 1W SME; C) fixation of both glass devices via the foam and D) lifting the locked objects.

Finally, a use case was investigated in which FOIM was considered as a possible joining technology. This time, FOIM was placed between a crystallizer bowl and a beaker (**Figure** **11**). Upon filling the beaker with boiling water, the foil started expanding. Remarkably, the beaker was fixed inside the crystallizer bowl within 5 min of cooling to 23 °C, which even allowed the glassware to be lifted.

Upon reheating to 60 °C, a simple disassembly was demonstrated, as the foam softened. This proved once again that concepts such as the release of internal stresses in the course of assembly, as known from 4D printing,^[^ ^28^ ^]^ can be combined well with the later softening of the shape memory polymer. This way, the possibilities for end‐of‐life and end‐of‐use concepts have been further expanded.

## 3 Conclusion

FOIM, the introduced shape memory polymer concept of a one‐time thermal switching from foil to foam, combines switchable molecular and macroscopic design in a programmable material approach. It offers several possibilities and advantages compared to standard foams. These include, among others,

1. space‐saving storage and transportation,
2. gradually adjustable property profiles upon step‐by‐step triggering of shape recovery,
3. its use as “thermal foaming” technology to fill even complex spaces,
4. the use for fixing fragile objects by interlocking them and
5. according to initial findings, a high degree of occupational safety.

The introduced process of “thermal foaming” differs from purely chemical foaming and from physical foaming, since only heat must be applied. It can be used to foam cavity structures in car interiors, in outer space, and as a packaging material when shipping fragile goods. The prospect of saving storage space for foams may be of significance for the polymer‐producing industry. Further benefits can be anticipated in the construction sector as a replacement of assembly foams, in insoles and soles for body‐temperature programming and comfort fitting,^[^ ^29^ ^]^ in medical applications, e. g. for the treatment of cerebral aneurysms,^[^ ^13^ ^]^ and in pharmaceutical applications as drug delivery systems.^[^ ^30^ ^]^ All these applications are focused on the thermoresponsive one‐way SME. Shape memory polymer foams containing magneto‐responsive fillers offer the prospect of expanding the spectrum, e.g. to applications in robotics.^[^ ^31^ ^]^ In any case, the physical, functional, and possibly also biological properties can be chemically adjusted to meet the technical requirements. The idea of producing thermally switchable semi‐finished products from shape memory polymer is now in the world and invites the development of further potential.

## 4 Experimental Section

### Synthesis

**Figure 12:**
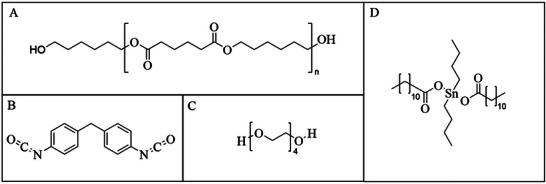
Chemicals used for the synthesis of the PEUU foam: A) Poly(1,6‐hexylene adipate) (PHA), B) 4,4’‐methyl‐diphenyl‐diisocyanate (MDI), C) polyethylene glycol (PEG 200) and D) dibutyltin dilaurate (DBTL).

Commercially available Diexter G240 (Chimica Organica Industriale Milanese S.p.A., Offanengo, Italy) was utilized as soft segment building block to synthesize the polyester urethane urea foam (PEUU foam). Diexter G240 is a linear poly(1,6‐hexylene adipate) diol (cf. **Figure** **12**A) with a molecular weight of 2970 g mol^−1^. 4,4’‐Methylene diphenyl diisocyanate (Covestro Deutschland AG, Leverkusen, Germany) (cf. Figure 12B), chain extended with PEG (Merck KGaA, Darmstadt, Germany; cf. Figure 12C) having a molecular weight of 200 g mol^−1^, was used to build up the hard segment phase. The ratio of NCO/OH was set to 1.06 with a hard segment content of 27 wt.%. Additionally, dibutyltin dilaurate (DBTL) (cf. Figure 12D) from Sigma–Aldrich (St. Louis, Missouri, United States of America) was employed as a catalyst, and water as blowing agent. To prepare the PEUU foam, following a reactive foaming procedure, the polyol was melted at 100 °C in a polypropylene beaker overnight and the isocyanate was melted in a separate vessel at 60 °C for 1 h. The polypropylene beaker with the polyol was thermally insulated using a polystyrene mold when taken out of the oven, and the polyol was thoroughly mixed with stabilizers, catalyst, cross‐linker, and the blowing agent. Afterward, the isocyanate was added while stirring vigorously to ensure good mixing. When the foam started rising, the beaker was removed from the stirrer, to enable a free rise of the foam. Before further processing and characterization, the foam was left to rest for 3 h at 60 °C and at least 24 h at 23 °C.

### Programming and Characterization of Shape Recoverability

The preparation of foil required thermomechanical treatment of the synthesized PEUU foam. After synthesis, the foam was cut into an appropriate sample size using a band saw (HBS 230 HQ, Holzmann Maschinen GmbH, Haslach, Austria) and a scalpel. Cube‐shaped samples of the pre‐cut foam, measuring ≈30 mm × 30 mm × 40 mm were then heated to 60 °C. After maintaining the temperature for 2 h, the foam was placed between two square metal plates with a side length of 120 mm and positioned in a manual press (Bernardo WK 10 TH, PWA Handelsgesellschaft mbH, Linz, Austria), where 15.6 MPa of compressive stress was applied to the outside of the frame. Foam and metal frame were then left to cool down to 23 °C.

Afterward, the load was removed, and a foil, the so‐called “FOIM” was obtained. In the following, the overall procedure of this thermomechanical treatment will be referred to as “programming”. To investigate the shape recoverability of the foam, the foil was exposed to 60 °C for 15 min. The height of the pristine foam (h_foam, pristine_), the height of the sample after being programmed to foil (h_foil_), and after triggering the 1W SME (h_foam, recovered_) were determined with a caliper gauge (Horex 2211216 caliper gauge digital 150 mm, Helios Preisser, Gammertingen, Germany). The shape recovery ratio (R_r_) was calculated according to Equation (1):

(1) $$R_{r}=\frac{h_{foam,recovered}}{h_{foam,pristine}}\times 100\%$$

The change in height from foil to foam (∆h) was calculated using Equation (2):

(2) $$\Delta h=\frac{h_{foam,recovered}}{h_{foil}}\times 100\%$$

### Transparency Tests After Programming

To investigate the transparency of the programmed foil, four samples with a lateral length of 40 mm and a height of 10, 20, 30, and 50 mm were prepared. The samples were programmed according to the procedure described above and then placed on a sheet of paper on which letters of the font “Times New Roman” were printed in black color in sizes 11, 9, 8, 7, and 5. The transparency of the foil was examined in artificial light with the naked eye while photos were taken.

### Determination of Solubility

A sample of 1.0 g PEUU foam was placed in 40 mL of DMF at room temperature. If the sample did not dissolve under stirring within 24 h, the temperature was raised to 80 °C for 4 h. In cases where the sample did not dissolve completely, its weight was finally determined after filtration and drying the residue at 60 °C in a vacuum oven. The method was employed to estimate the extent of chemical cross‐linking in the material.

### Microscopic Imaging

To evaluate the structure of the foam including the size of its cells, microscopic images were taken with a Levenhuk DTX 90 (Levenhuk Zoom & Joy, Prague Poland) using the software application MicroCapture Plus. In addition, the surface structure was examined with a focus variation microscope (ConfoSurf CLV150, Confovis GmbH, Jena, Germany). Microscopic imaging was also employed to investigate the foil (FOIM) after programming and the recovered foam after triggering the 1W SME.

### Determination of Density and Open‐Cell Content

The density of the PEUU foam was determined with a Kern AEJ100 analytical balance (Kern & Sohn, Lörrach, Germany). For this purpose, three cube‐shaped samples with a lateral length of 20 mm were cut from the pristine PEUU foam using a scalpel and a band saw. The density was calculated from Equation (3).

(3) $$\rho =\frac{m_{dry}\;}{\mathrm{length}\;\times \mathrm{width}\;\times \mathrm{height}\;}$$

An in‐house procedure was used to quantify the open cell content.^[^ ^20^, ^32^ ^]^ The dimensions of three samples were determined together with the mass of the dry samples m_dry_. The cubes were then soaked for 72 h in silicon oil (Ebesil Öl B 0,65, Quax GmbH, Otzberg, Germany; η = 0.65 mm^2^ s^−1^; ρ = 0.76 g cm^−3^). A YBD‐03 density set (Kern & Sohn GmbH, Lörrach, Germany) was used to measure the buoyancy (m_Buoyancy_) of the samples to calculate the relative open cell content (OC_rel_).

### Determination of Heat Transfer

To compare the material properties of pristine PEUU foam before and after programming and triggering the 1W SME, the surface temperature of the foam opposite from the heating plate was measured using an InfraTec thermography system (VarioCAM HD, InfraTec GmbH, Dresden, Germany) with the respective software IRBIS 3 plus (InfraTec GmbH, Dresden, Germany). Three samples (40 mm × 40 mm × 10 mm) were cut with a scalpel and a bandsaw and placed on a heating plate, which was preheated to 100 °C. The surface temperature of the upper side of any sample was then measured for 30 min. The last average value of the surface temperature was reported. The sample was then programmed following the procedure described above. Afterward, the programmed sample was placed in an oven at 60 °C for 30 min to ensure complete shape recovery. Adjacently, the sample was left to cool to room temperature in a desiccator for 30 min and the thermographic measurement was repeated. The results before programming and after shape recovery were compared with each other.

### Differential Scanning Calorimetry

To investigate the thermal properties of the PEUU foam, including the on‐ and offset and the peak temperature of the melting and crystallization transition and the corresponding enthalpies, differential scanning calorimetry was performed with a TA DSC Q250 (TA Instruments, New Castle, USA). The measurement procedure included two cycles of heating to 100 °C and cooling to −70 °C with a rate of 10 °C min^−1^ and a waiting time of 2 min between each heating and cooling step. The phase transitions were characterized from the second cycle. To evaluate the crystallization behavior, the programmed foil (FOIM) was rapidly cooled to −80 °C, before being heated to 60 °C and cooled to −70 °C with rates of 1 °C min^−1^ and a waiting time of 2 min between each heating and cooling step.

### Dynamic Mechanical Analysis

The thermomechanical properties of the pristine PEUU foam were determined via dynamic mechanical analysis using a DMA Q800 (TA Instruments, New Castle, USA) equipped with compression clamps. Measurements were conducted on cube‐shaped samples with a lateral length of 10 mm. The stress‐deformation property was investigated according to DIN EN ISO 3386.^[^ ^18^, ^19^ ^]^ For this purpose, the foam was compressed by 50% at 23 °C with a loading rate of 100% min^−1^ before the resulting stress was released. This loading/unloading cycle was repeated three times. Subsequently, a compression of 40% with a loading rate of 100% min^−1^ was applied and the required stress (CC_40_) was determined for three different samples.

In another experiment, three consecutive measurement cycles were carried out to evaluate the compressive stress–strain behavior. The foam was always compressed by 60% at a load rate of 50% min^−1^ at 23 °C before being unloaded at the same rate.

To characterize the thermomechanical properties of PEUU foam and foil the compression clamps were replaced by film clamps. The sample size of the foam was 10 mm × 10 mm × 2 mm and that of the foil 10 mm × 10 mm × 1 mm.

One thermal cycle with a frequency of 10 Hz, a static force of 0.5 N, and a vibration amplitude of 10 µm was carried out to characterize the thermomechanical properties of the materials. The sample was first cooled to −80 °C and the temperature kept constant for 30 min. It was then heated to 100 °C with a rate of 3 °C min^−1^. The maximum of the loss modulus E’’ and the loss factor tan δ were used to determine the glass transition temperature (T_g_).

### Ball Rebound Testing

A ball rebound‐tester (Barreis Prüfgerätebau GmbH, Oberdischingen, Germany) was utilized to determine the elasticity of the pristine foam, the programmed foam, and the partially as well as the fully recovered foam. A metal ball was inserted into a down pipe having a height of 500 mm and the rebound of the ball was measured. This procedure was repeated five times, and the mean value was determined. To trigger a gradual shape recovery, the sample was subjected to 40, 50, 60, 70, 80, 90, and 100 °C for 30 min each. The measured values were averaged.

### Fourier‐Transform Infrared Spectroscopy

The pristine foam and the programmed foil were investigated by Fourier Transform Infrared (FT‐IR) spectroscopy with a Nicolet Nexus 470/670/870 FT‐IR spectrometer (Thermo Fisher Scientific, Madison, WI, USA). Using an attenuated total reflectance (ATR) device, samples were scanned for a total of 100 scans with a spectral resolution of 2 cm^−1^ to obtain an average spectrum from 4000 to 650 cm^−1^.

### Demonstrator Development

The foamable cavity structures used for demonstration purposes were cut from acrylic glass with a 30 W laser cutter (Epilog Laser, Golden, CO, USA). The cutting pattern was created with CorelDraw 2019 (Corel Corp., Ottawa, ON, Canada) and is displayed in Figure S6 (Supporting Information). The acrylic glass was glued together using an instant adhesive (Pattex Repair 60s, Henkel, Düsseldorf, Germany) and left to fully dry for 24 h. The foam was cut with a scalpel, and a band saw to a standard sample size (120 mm × 50 mm × 40 mm). The samples were programmed, following the general procedure as described in the Section “Programming and Characterization of Shape Recoverability”. First, the samples were tempered at 60 °C for 60 min and then compressed using the manual press and a load of 3 t. After cooling to room temperature, the force was removed, and the samples were inserted in the demonstration device.

## Conflict of Interest

The authors declare no conflict of interest.

## Author Contributions

Conceptualization was performed by A.P. and T.P.; Formal analysis was performed by A.P.; Funding acquisition was performed by T.P.; Investigation was performed by A.P.; Methodology was performed by A.P.; Project administration was performed by A.P. and T.P.; Supervision was performed by T.P.; Validation was performed by A.P.; Original draft was written by A.P.; Review and editing by T.P. and A.P. All authors have read and agreed to the published version of the manuscript.

## Supporting information



Supporting Information

Supplemental Video 1

## Data Availability

The data that support the findings of this study are available from the corresponding author upon reasonable request.
