# Supplementary material for: FOIM: Thermal Foaming of Shape Memory Polyurethane Foil
Source: Macromol Rapid Commun. 2025 Feb 6;46(8):2401103. doi: 10.1002/marc.202401103 (PMC12004890; doi:10.1002/marc.202401103)
Supplement: Supplementary file 1 — Supporting Information [file MARC-46-2401103-s002.docx]

Supporting Information

FOIM: Thermal Foaming of Shape Memory Polyurethane Foil

Anna-Lisa Poser, Thorsten Pretsch*


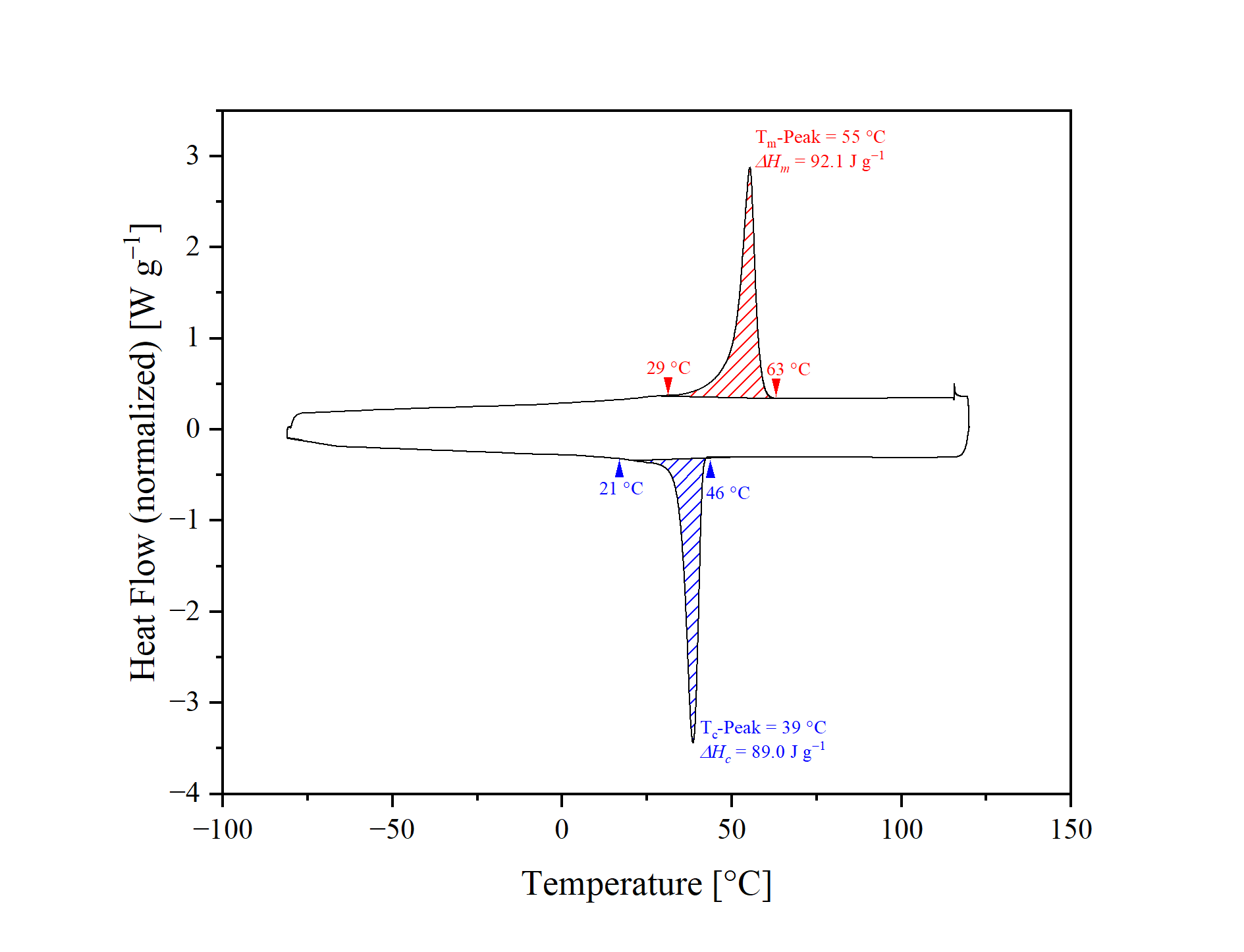


Figure S1: DSC thermogram of the pure PHA diol showing the second heating (PHA melting signal in red color) and cooling (PHA crystallization signal in blue color) with rates of 10 °C min^−1^.


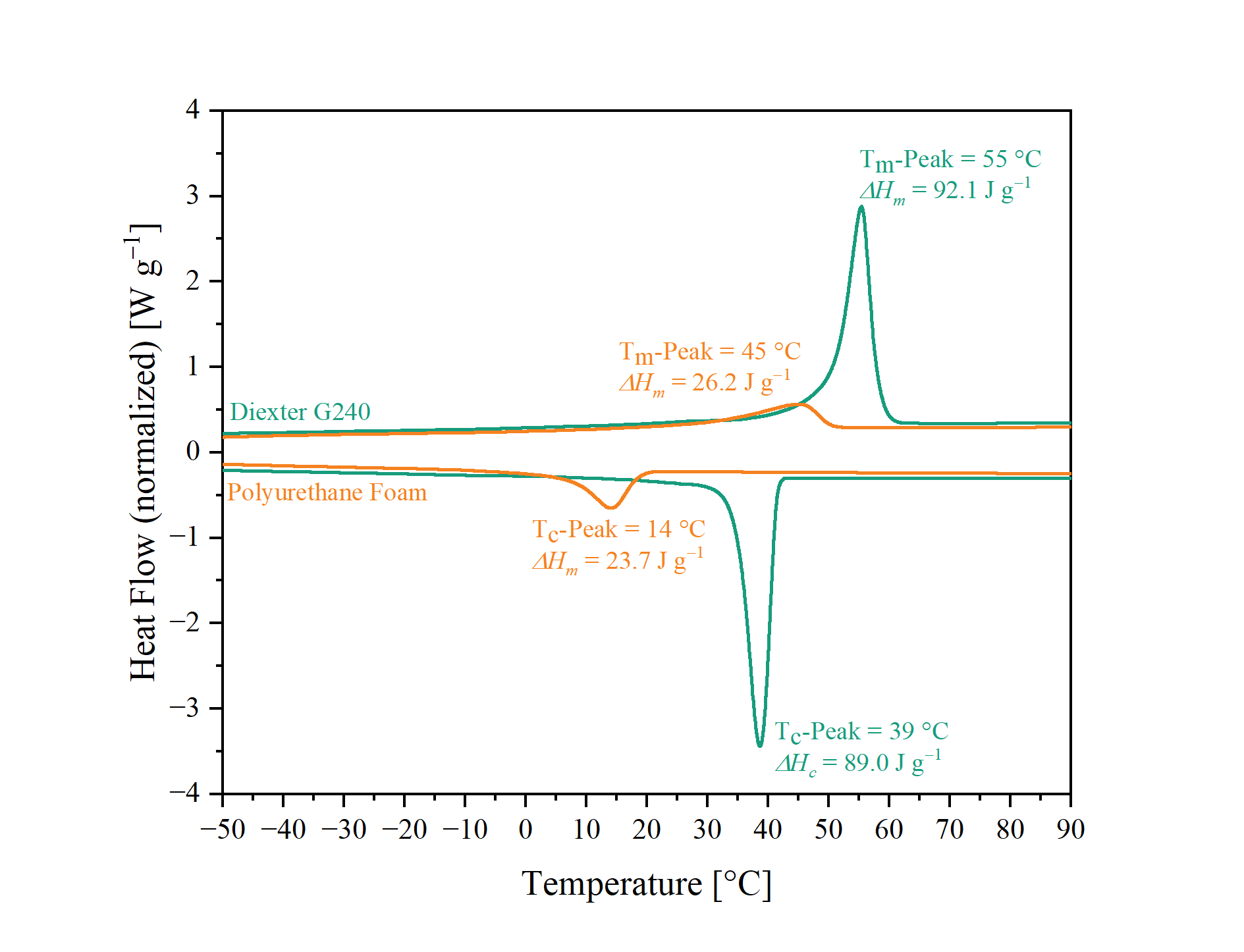


Figure S2: DSC thermograms of PHA diol (in green color) and PEUU foam (in orange color). The data exhibits the second thermal cycle with heating and cooling rates of 10 °C min^−1^.


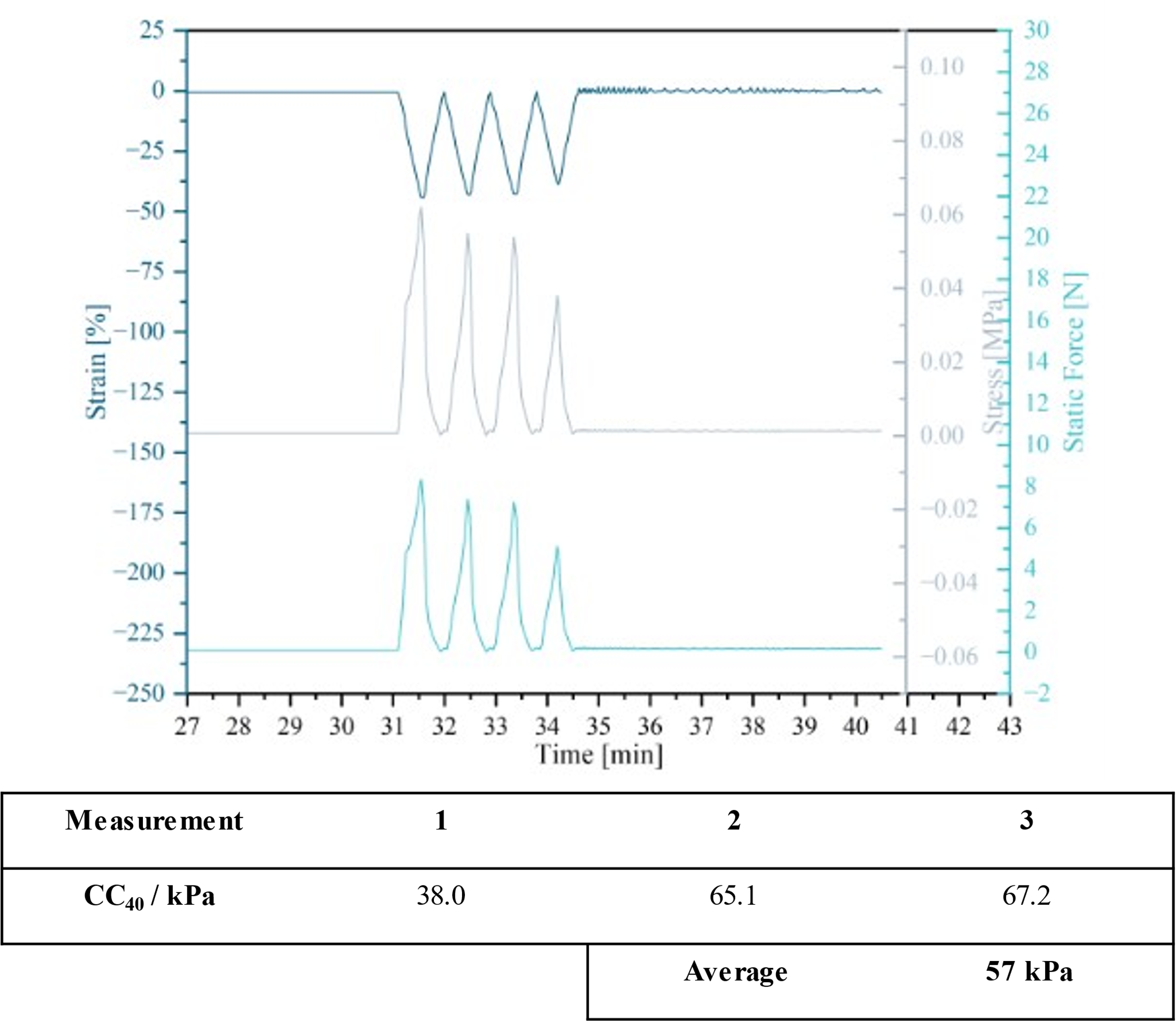


Figure S3: Measurement of the stress-deformation property of the pristine foam in a threefold measurement.


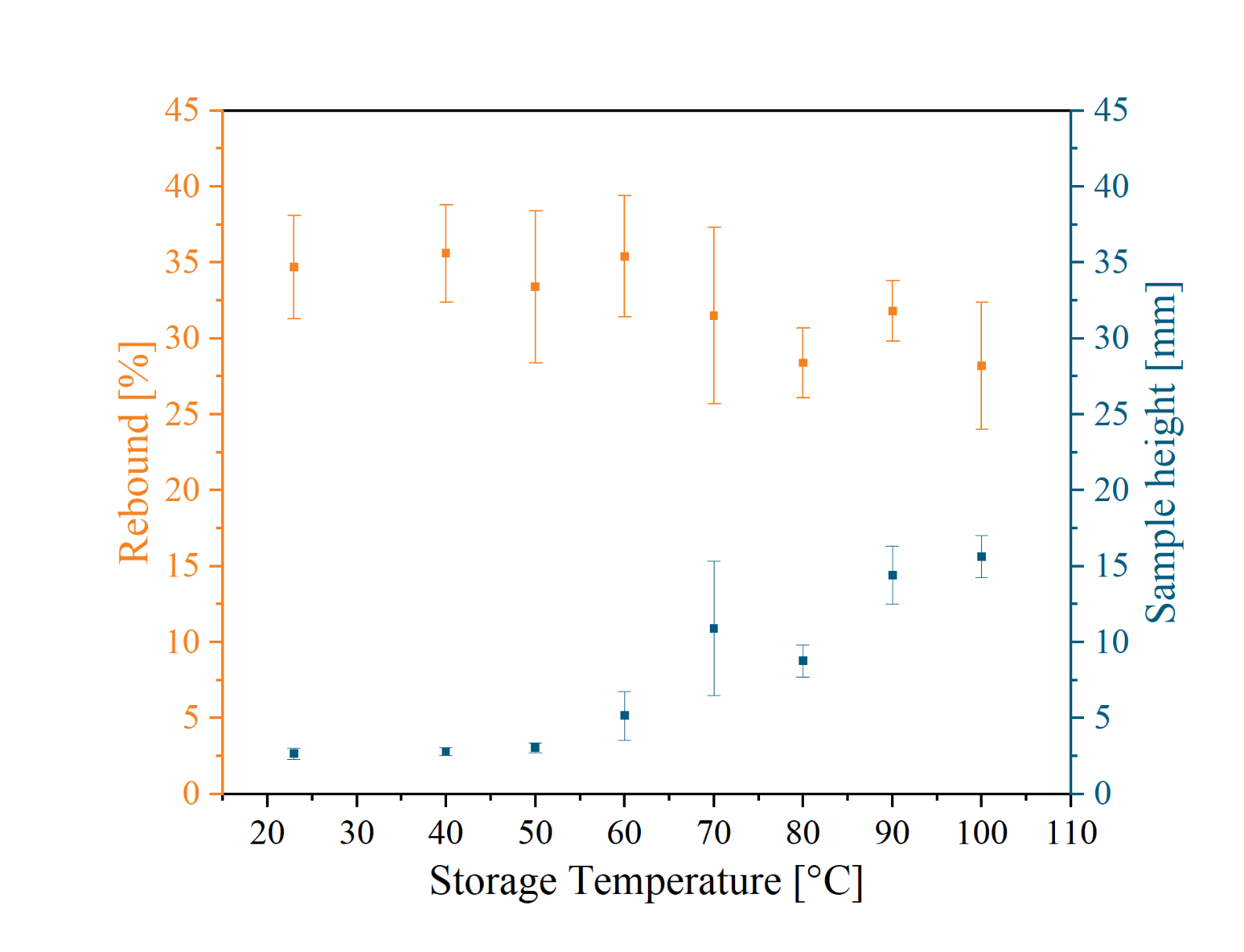


Figure S4: Characterization of FOIM regarding its ball rebound properties at room temperature after 30 min storage at different temperatures.


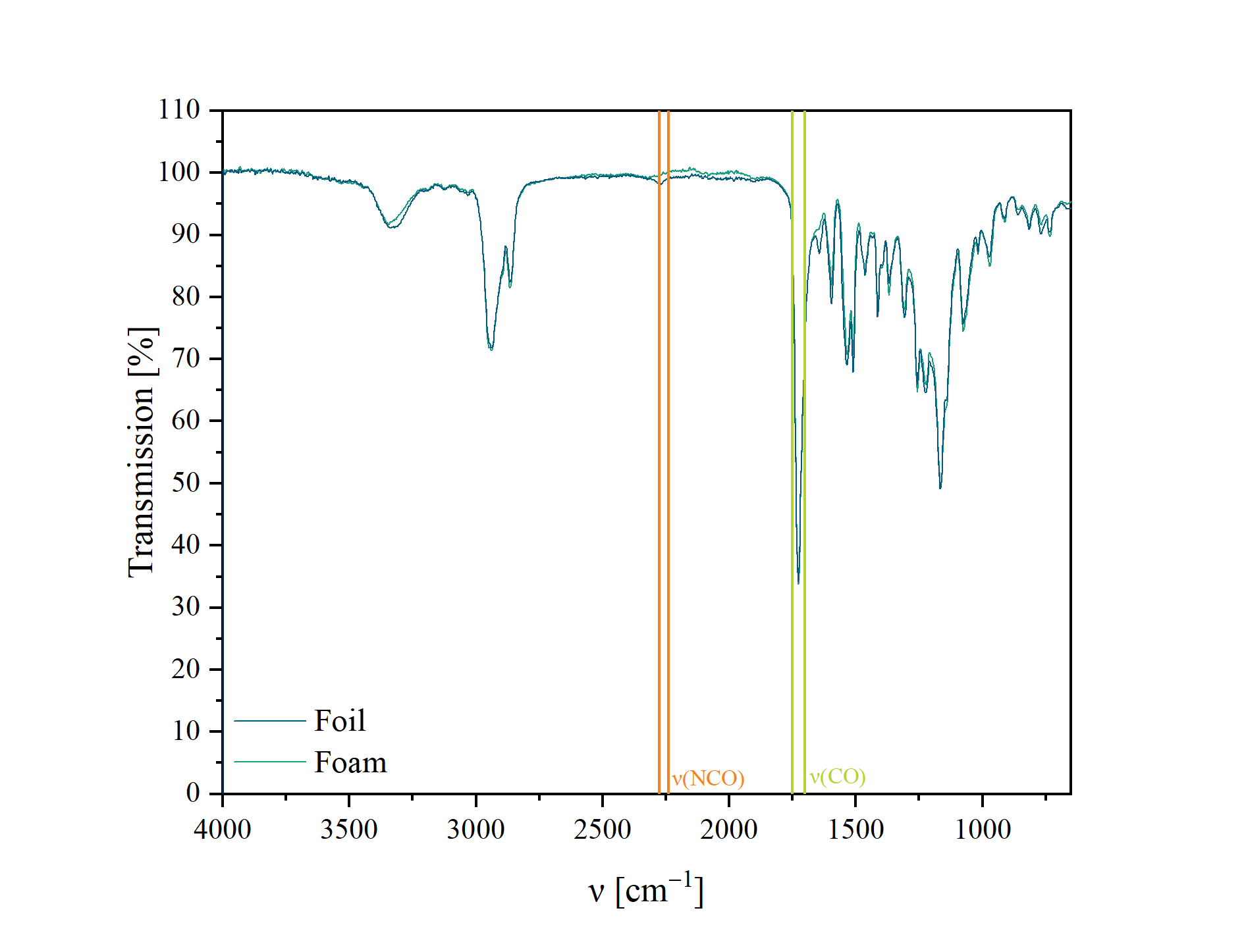


Figure S5: FT-IR spectra of PEUU foam and the same sample after programming to foil. Regions, in which stretching vibrations of terminal isocyanate groups were expected, but not detected, are drawn in orange color. Regions, in which stretching vibrations of “free” non-hydrogen-bonded (dominating signal) and hydrogen-bonded carbonyl groups (broad shoulder) were assigned, are drawn in green color.^[1]^


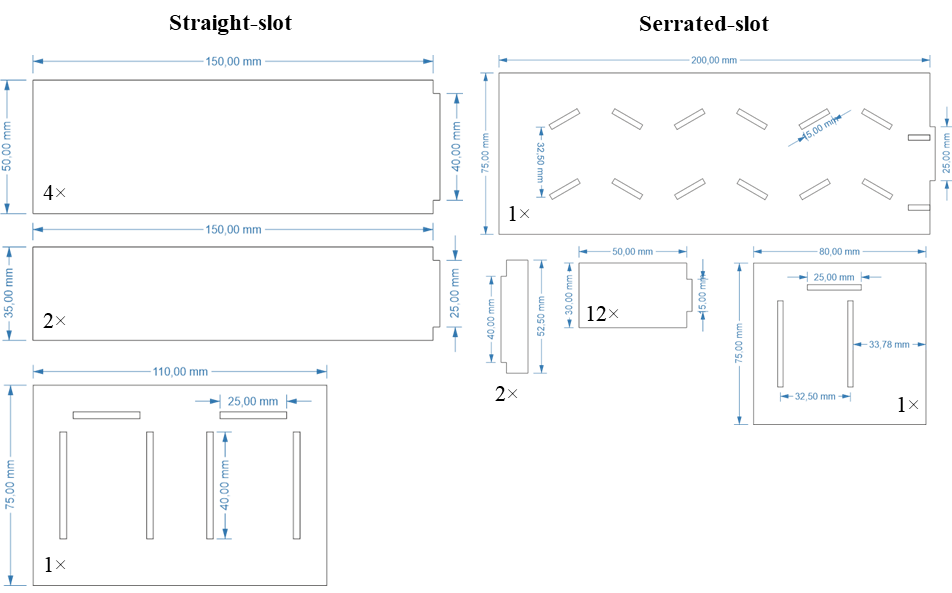


Figure S6: Dimensioning of acrylic glass parts as used to produce foamable cavity structures.

A video (Video S1) on thermal foaming using FOIM can be accessed.

# Reference

[1] J. Coates, *Encyclopedia of Analytical Chemistry.* **2000**, 10815.
